# Trajectories of cognitive function and frailty in older adults in China: a longitudinal study

**DOI:** 10.3389/fnagi.2024.1465914

**Published:** 2024-11-14

**Authors:** Xiaoyi Ji, Yue Wu, Zijie Gu, Zhujun Zhong, Kerui Wang, Suni Ye, Yang Wan, Peiyuan Qiu

**Affiliations:** ^1^West China School of Public Health and West China Fourth Hospital, Sichuan University, Chengdu, China; ^2^Hangzhou Seventh People’s Hospital, Zhejiang University School of Medicine, Zhejiang, Hangzhou, China

**Keywords:** cognitive function, frailty, dual trajectories, older adults, aging

## Abstract

**Background:**

Cognitive impairment and frailty are common issues in older adults. Understanding the co-development trajectories of these conditions can provide valuable sights for early detection and intervention in high-risk individuals.

**Objectives:**

This study aims to identify the co-development of cognitive function and frailty and explore the associated characteristics.

**Methods:**

We analyzed data from 8,418 individuals aged 55 years and above who participated in the China Health and Retirement Longitudinal Survey between 2011 and 2018. Group-based dual trajectory modeling and logistic regression were used to identify trajectory groups and assess associations with risk factors.

**Results:**

Two distinct dual trajectories were identified: “Consistently Robust” group (76.12%) and “Consistently Severe” group (23.88%). Factors such as being female, older age, lower levels of education, residing in rural areas, being unmarried, and having comorbidities such as hypertension, diabetes, complete tooth loss, vision impairment, or hearing impairment were associated with a higher likelihood of being assigned to the “Consistently Severe” group.

**Conclusion:**

Our findings suggest a co-development pattern between cognitive function and frailty in Chinese older adults aged 55 years and above. While cognitive impairment may be irreversible, frailty is a condition that can be potentially reversed. Early detecting is crucial in preventing cognitive decline, considering the shared trajectory of these conditions.

## 1 Introduction

China’s population is aging rapidly. By the end of 2022, the number of individuals aged 60 and above had reached 280.04 million, accounting for approximately 19.8% of the total population (Affairs MoC, 2023). With this demographic shift, the prevalence of cognitive impairment and frailty among older adults has increased, significantly affecting their quality of life and placing a considerable economic and caregiving burden on their families.

A growing body of research has explored the relationship between frailty and cognitive impairment in older adults. For example, a longitudinal study conducted in South Korea among older adults aged 65 and above found that individuals who remained frail or transitioned from non-frail to frail had higher likelihood of lower cognitive function (Nari et al., 2021). Similarly, a prospective study in Japan revealed that frailty was associated with cognitive decline in older adults over a two-year period (Chen et al., 2018). These findings are further supported by several studies demonstrating that frail older adults are at greater risk of experiencing cognitive impairment compared to their non-frail counterparts (Li et al., 2023; Kim et al., 2014). Conversely, there is evidence that cognitive decline also serves as a risk factor for developing frailty (Mulero et al., 2011; Doba et al., 2012; Yuan et al., 2021). For example, Li et al. (2023) study revealed that older adults with cognitive impairment have an estimated 19.5% higher one-year incidence of frailty compared to those without cognitive impairment (Belleville et al., 2022).

Despite substantial evidence, the causal relationship between frailty and cognitive impairment remains complex and not fully understood. A review by Robertson et al. (2013) discusses how frailty and cognitive decline interact within a cycle of age-associated decline, suggesting a bidirectional nature of this relationship. Furthermore, some researchers propose that frailty and cognitive impairment may develop simultaneously, sharing common biological causes (Buchman et al., 2007; Buchman et al., 2014). The oxidative stress theory suggests that the brain’s vulnerability to oxidative damage can lead to cognitive impairment (Mulero et al., 2011), while reactive oxygen species contribute to frailty (Kregel and Zhang, 2007). As a result, researchers are starting to investigate the co-occurrence of cognitive decline and frailty at the population level. Group-based dual trajectory modeling (GBDTM) has emerged as a powerful analytical tool for examining these parallel developments, allowing for a better understanding of how frailty and cognitive impairment influence each other over time.

GBDTM is an extension of Nagin’s Group-Based Trajectory Model (GBTM). While GBTM assumes that the population consists of multiple unobserved subgroups, each with its own trajectory, and uses finite mixture modeling to estimate these groups, GBDTM focuses on modeling the developmental trajectories of two related outcomes (Nagin and Odgers, 2010). It enhances GBTM by incorporating polynomial models of time (age) and using maximum likelihood estimation to assign individuals to subgroups (Nagin and Tremblay, 2001). Unlike GBTM, GBDTM allows for statistical associations between trajectories, enabling the exploration of their co-relationships. It employs simultaneous models to estimate the trajectories of two variables, with relationships that can vary across subgroups. These relationships can be linear or nonlinear, capturing the complex dynamics between variables. A key strength of GBDTM is its ability to identify natural subgroups within a population based on joint changes in the two variables, with these subgroups emerging from the data rather than being predefined (Nagin and Tremblay, 2001; Muthén and Muthén, 2000; Curran and Hussong, 2003).

GBDTM has proven effective in identifying dual trajectories, making it well-suited for exploring common trends in cognitive function and frailty over time. For example, a longitudinal study in the U.S. showed that 20% of older adults exhibited concurrent trajectories of “persistent frailty” and “persistent severe cognitive impairment” (Yuan et al., 2022). Similarly, another study identified four distinct trajectories of frailty and cognitive functioning, with 6.5% of older adults showing a pattern of cognitive frailty (Liu et al., 2018). In Mexico, a longitudinal study revealed that 63% of older adults with an increasing frailty index experienced rapid cognitive decline, while 68% of those with no frailty changes maintained cognitive stability (Howrey et al., 2020). In Milan, 4.3% of old adults showed continuous decline in both cognitive and physical functioning (Ferraro et al., 2021). These studies, based on GBDTM, highlight the common trajectories of frailty and cognitive impairment, though the patterns vary across countries.

While some studies have been conducted in Europe and America, our research focuses on a population that has been underrepresented in the literature. The unique cultural, lifestyle, and demographic factors in China may lead to different dual trajectories of cognitive function and frailty compared to those observed in Western populations. By exploring this specific context, we aim to provide valuable insights into how these dual trajectories manifest and to identify distinct associated factors. Our findings could help determine whether these dual trajectories are universal or context-specific, ultimately contribute to a more comprehensive understanding of cognitive and frailty dynamics.

## 2 Materials and methods

### 2.1 Data

The data for this study were obtained from the China Health and Retirement Longitudinal Study (CHARLS), which is a large-scale interdisciplinary longitudinal survey project conducted by the National Development Research Institute of Peking University and implemented by the China Social Science Survey Center of Peking University. The baseline survey was conducted in 2011, covering 150 counties and 450 communities (villages) across 28 provinces, autonomous regions, and municipalities in China, targeting individuals aged 45 and above. Subsequent nationwide follow-up surveys were conducted in 2013, 2015, 2018 and 2020.

This study used data from the 2011 CHARLS cohort, along with newly recruited participants in 2013 as the baseline, resulting in a total of 21,131 individuals. The inclusion criteria for the study were: (1) participants aged 55 and above at baseline; and (2) participants who completed at least two follow-up waves in 2013, 2015, and 2018. The exclusion criteria included: (1) missing data for key variables. After excluding participants younger than 55 years old, 11,210 participants remained. Further exclusion of individuals with missing key variables resulted in a final sample of 8,814 older adults leading to an attrition rate of 21.37%.

### 2.2 Measures

#### 2.2.1 Cognitive function

In this study, we focused on the memory dimension to ensure consistent measurement of cognitive function from 2011 to 2018. Memory was assessed using both immediate memory and delayed memory tests (Yuan et al., 2022). Immediate memory involved presenting participants with a set of 10 words, which they were then asked to recall within a two-minute period with scores ranging from 0 to 10. Delayed memory was evaluated by assessing participants’ recall of the same 10 words after testing their depression, calculation ability, and visuospatial ability. Scores for delayed memory also ranged from 0 to 10. Therefore, the combined score for immediate and delayed memory ranged from 0 to 20, with lower scores indicating poorer cognitive function.

#### 2.2.2 Frailty

The frailty status of the individuals was assessed using the Frailty Index (FI), which evaluates the degree of frailty by calculating the cumulative number or proportion of health deficits. This is typically represented as the ratio of cumulative health deficit count to the total number of health items included, with total score ranging from 0 to 1; higher scores indicating a more severe frailty status (Liu et al., 2018). In this study, the FI included 38 health indicators, including 6 activities of daily living (ADL), five instrumental activities of daily living (IADL), nine physical function limitations, 12 chronic diseases, five mental health indicators, and one self-rated health indicator (Supplementary Table 1).

#### 2.2.3 Covariates

We included several covariates in our analysis, including age, gender, residence, marital status, education, annual household expenditure, chronic disease, current drinking status, visual and hearing impairment, and complete tooth loss. Annual personal expenditure was categorized as “Lower than the average” and “Higher than the average” in comparison to the annual personal expenditure of Chinese residents in 2010. Vision impairment was defined as meeting one of the following criteria: self-reported poor vision, near vision impairment, or distance vision impairment (Howrey et al., 2020). Hearing impairment was defined as meeting one of the following criteria: having problems with deafness or partial deafness, wearing a hearing aid (Ferraro et al., 2021), or self-reporting poor hearing (Nagin, 1999). The coding of variables is presented in Supplementary Table 2.

### 2.3 Statistical analysis

We provided an overview of the participants’ baseline characteristics, as well as their cognitive function and frailty status for each wave. We then conducted trajectory analysis, which involved the following steps:

First, we performed separate group-based trajectory modeling (GBTM) for cognitive function and frailty. GBTM identifies unobserved heterogeneous subgroups within the sample population and develops trajectories based on their trends (Nagin, 1999). Individuals are assigned to the most likely subgroups based on the largest posterior probability (Huang et al., 2013).

Second, we used group-based dual trajectory modeling (GBDTM) for cognitive function and frailty, treated these as dependent variables with year as the time variable. A dual development trajectory was fitted to assign similar individuals to different subgroups. To determine the most appropriate number of trajectories for GBDTM, we tested separate GBTM for cognitive function and frailty with two or more trajectory groups. In GBDTM, the number of dual trajectory groups and slope parameters in each group were set according to the results from GBTM.

We evaluate model fit using the Bayesian Information Criterion (BIC), entropy, the smallest group size (SG%) including at least 5% of the sample, and an average posterior probability of assignment (APPA) >0.70.

Finally, logistic regression analyses were conducted to determine the association between the potential associated factors and the dual trajectories of cognitive function and frailty. Group differences were considered significant if *p* < 0.05 (two- tailed).

## 3 Results

### 3.1 Sociodemographic and health characteristics

In this study, a total of 8,814 participants were included, with a mean age of 62.6 (SD±6.5) years. Among them, males accounted for 50.52 and 49.48% for females. Most participants were literate (71.24%), residing in rural areas (62.29%), married (87.12%). Only 11.04% of the participants had annual personal expenditure higher than the average. And 66.38% of the participants reported never drinking alcohol. In terms of health conditions, with regard to missing data, at least 27.39% had hypertension, 6.51% had diabetes, 10.40% had experienced complete tooth loss, 43.65% had vision impairment, 17.57% had hearing impairment (Table 1).

**Table 1:** Baseline characteristics distribution of participants (*N* = 8,814).

	Characteristics	Number of participants (*n*)	Percentages (%)
Gender	Male	4,453	50.52
	Female	4,361	49.48
Age	55–64	5,937	67.36
	65–74	2,353	26.70
	≥75	524	5.94
Education	Illiterate	2,535	28.76
	Literate	6,278	71.23
	Missing	1	0.01
Residence	Rural	5,490	62.29
	Urban	3,324	37.71
Marriage	Unmarried	1,135	12.88
	Married	7,679	87.12
Annual personal expenditure (compare to the average)	Lower	7,841	88.96
	Higher	973	11.04
Drinking	Still drinking	2,316	26.28
	Abstinence	647	7.34
	Never drinking	5,850	66.37
	Missing	1	0.01
Hypertension	No	5,445	61.78
	Yes	2054	23.30
	Missing	1,315	14.92
Diabetes	No	6,989	79.29
	Yes	487	5.53
	Missing	1,338	15.18
Complete tooth loss	No	6,751	76.59
	Yes	784	8.89
	Missing	1,279	14.51
Vision impairment	No	4,503	51.09
	Yes	3,488	39.57
	Missing	823	9.34
Hearing impairment	No	7,262	82.39
	Yes	1,548	17.56
	Missing	4	0.05

**Table 2:** Model search process in GBTM models of cognitive function and frailty.

Number of trajectories	Cognitive function				Number of trajectories	Frailty			
	BIC	APPA	SG%	Entropy		BIC	APPA	SG%	Entropy
GBTM									
2	−77040.69	>0.91	49.77	0.71	2	34960.52 37612.81	>0.94	23.16	0.91
3	−76291.91	>0.83	21.60	0.68	3		>0.91	7.41	0.88
4	−76063.55	>0.79	11.07	0.66	4	38838.85	>0.87	2.67	0.86
5	−75951.43	>0.65	8.07	0.65	5	39259.81	>0.84	1.56	0.83

Final number of trajectory groups was determined				
GBDTM				
Number of dual trajectory groups	BIC	APPA	SG%	Entropy
2	−44961.65	>0.94	24.12	0.90
3	−42195.22	>0.91	7.69%	0.88
4	−40279.65	>0.86	4.37%	0.81

BIC, Bayesian information criteria; APPA, average posterior probability of assignment; SG%, the smallest group %.

### 3.2 Identifying dual trajectories of cognitive function and frailty among Chinese older adults

In the GBDTM models, we determine the number of dual trajectory groups and the slope parameters for each group based on the outcomes derived from the GBTM models for both cognitive function and frailty (Table 2) (Liu et al., 2018). Two dual trajectories were identified and labeled as follows (Supplementary Table 3): “Consistently Robust” group [Group 1 (G1); 76.12%] and “Consistently Severe” group [Group 2 (G2); 23.88%]. G1 demonstrated a high initial value and stable trend in cognitive function (slope = −0.29, *p* < 0.001), a low initial value and stable trend in frailty (slope = 0.00, *p* < 0.05) over time. Whereas G2 demonstrated a decline in cognitive function (slope = −0.30, *p* < 0.001) and an increase in frailty level (slope = 0.01, *p* < 0.001).

**Figure 1:**
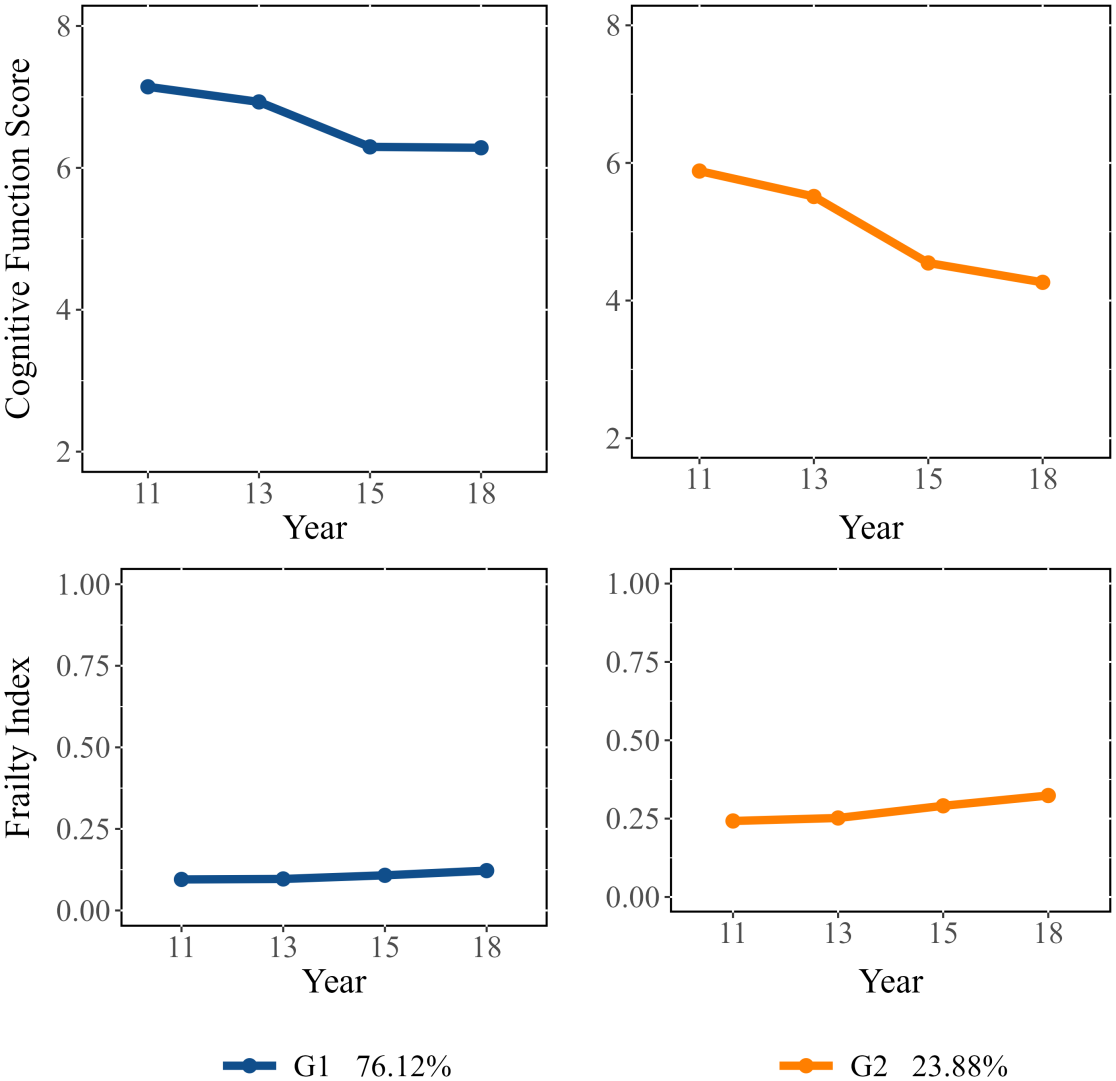
Dual trajectories of cognitive function (represented by cognitive function score) and frailty (represented by frailty index).

Figure 1 illustrates the levels and shapes of change of these dual trajectories. Participants in G1 exhibited better cognitive function than those in G2, despite a decline over time. By the end of the follow-up, G1 participants maintained relatively stable cognitive and were physically robust throughout the study. In contrast, participants in G2 experienced a continuous deterioration in both frailty and cognitive function, with changes occurring at a greater magnitude than in G1.

### 3.3 Examining associated factors of identified dual trajectories of cognitive function and frailty

Multivariate analyses of dual trajectories indicated that participants aged 65 to 74 at baseline were 1.71 times more likely to experience deteriorating cognition and frailty [OR: 1.71; 95%CI: 1.51–1.93]. Those aged 75 and older had 1.66 times higher likelihood of falling into the “Consistently Severe” category [OR: 1.66; 95%CI: 1.32–2.08]. Women were also at increased risk, with a likelihood 1.59 times greater [OR: 1.59; 95%CI: 1.39–1.81]. Conversely, literate individuals had a lower probability of being assigned to the “Consistently Severe” group [OR: 0.72; 95%CI: 0.63–0.82]. Similarly, those living in urban areas [OR: 0.61; 95%CI: 0.54–0.69] and those who were married [OR: 0.74; 95%CI: 0.63–0.87] also showed lower likelihoods of experiencing continuous cognitive and frailty decline.

Additionally, older adults with chronic diseases at baseline were more likely to be in the “Consistently Severe” group. Specifically, participants with hypertension were 2.59 times more likely to experience worsening cognition and frailty [OR: 2.59; 95%CI: 2.30–2.92], those with diabetes were 2.43 times more likely [OR: 2.43; 95%CI: 1.98–2.97], and participants with vision impairment were 2.02 times more likely [OR: 2.02; 95%CI: 1.80–2.26]. Furthermore, the probability of being assigned to the “Consistently Severe” group was 30% higher [OR: 1.30; 95%CI: 1.09–1.54] for individuals with complete tooth loss compared to those without, and 69% higher [OR: 1.69; 95%CI: 1.47–1.93] for those with hearing impairment.

Above results can be found in Table 3. Additionally, the results of the logistic regression analyses conducted separately for cognitive function and frailty can be found in Supplementary Tables 4, 5.

**Table 3:** Associated factors of dual trajectories.

Variables	Group 2/Group 1 (ref)	
	OR	95% CI
Age		
55–64 (ref)		
65–74	1.71***	1.51–1.93
≥75	1.66***	1.32–2.08
Education		
Illiterate (ref)		
Literate	0.72***	0.63–0.82
Gender		
Male (ref)		
Female	1.59***	1.39–1.81
Residence		
Rural (ref)		
Urban	0.61***	0.54–0.69
Average annual personal expenditure		
Low (ref)		
High	1.11	0.93–1.33
Marriage		
Unmarried (ref)		
Married	0.74***	0.63–0.87
Drinking		
Still drinking (ref)		
Abstinence	0.93	0.71–1.20
Never drinking	1.43***	1.22–1.66
Hypertension		
No (ref)		
Yes	2.59***	2.30–2.92
Diabetes		
No (ref)		
Yes	2.43***	1.98–2.97
Complete tooth loss		
No (ref)		
Yes	1.30**	1.09–1.54
Vision impairment		
No (ref)		
Yes	2.02***	1.80–2.26
Hearing impairment		
No (ref)		
Yes	1.69***	1.47–1.93

OR, odds ratio; 95% CI, 95% confidence interval; ref, reference group; *p < 0.05; **p < 0.01; ***p < 0.001.

## 4 Discussion

In this longitudinal study, we observed two distinct dual trajectories of cognitive function and frailty among older adults in China. One group exhibited better cognitive function and lower levels of frailty compared to the other group. This finding is consistent with previous studies conducted in both institutionalized (Yuan et al., 2022) and non-institutionalized (Howrey et al., 2020) American older adults, which also identified consistent developmental trends in cognitive function and frailty. The correlation between changes in cognitive function and frailty may contributed to common underlying mechanisms. Previous research has highlighted the influence of vascular changes, hormones, vitamin D levels, inflammation, insulin resistance, and nutrition on both cognitive function and frailty in older individuals (Halil et al., 2015). Moreover, dysregulated HPA stress response, imbalanced energy metabolism, mitochondrial dysfunction, oxidative stress, and neuroendocrine dysfunction have been proposed as shared etiological factors for the concurrent occurrence of frailty and cognitive decline (Ma and Chan, 2020). This consensus was reflected in a 2013 conference held by the International Association of Nutrition and Aging and the International Association of Gerontology and Geriatrics, which coined the term “cognitive frailty” to describe the coexistence of cognitive impairment and frailty (Kelaiditi et al., 2013). Identifying individuals at a high risk of experiencing rapid declines in cognitive function and escalating frailty is crucial, given the heightened threat of mortality and disability associated with this condition (Ma et al., 2021).

We observed that certain characteristics, such as being female, older, illiterate, residing in rural areas, and unmarried were more prevalent among individuals assigned to the “Consistently Severe” group. These individuals also displayed worse overall health compared to those in the “Consistently Robust” group, including a higher prevalence of conditions such as diabetes, hypertension, hearing impairment, vision impairment, and complete tooth loss. The association between increasing age and being assigned to the “Consistently Severe” group is likely attributable to the natural deterioration of organ functions with aging. On the other hand, married older adults were more likely to be categorized into the “Consistently Robust” group, possibly due to their engagement in social activities and greater social support. We found that older adults residing in rural areas had a higher likelihood of being assigned to the “Consistently Severe” group, which aligns with findings from other research (Liu et al., 2021). However, we did not find a significant impact of alcohol consumption on cognitive function changes in older adults. The relationship between alcohol consumption and cognition remains inconclusive. While some studies suggest a connection (Fein et al., 2006; Montejo and Rico-Villademoros, 2008; García-Marchena et al., 2020), our findings are in line with the results of Linglong et al. (2021), who found no impact of alcohol consumption on different cognitive function trajectories among adults aged 65 and above in China. To further investigate this association accurately, future research is recommended to include additional specific variables related to alcohol consumption, such as the type of alcohol consumed, dosage, and other relevant factors.

Identifying potential factors that contribute to assigning individuals into high-risk groups allows for targeted interventions aimed at delaying cognitive function decline and frailty. In our research, we identified modifiable health factors suitable for intervention, including hypertension, diabetes, visual impairment, hearing impairment and complete tooth loss. Previous studies have suggested an association between hypertension and both cognitive function and frailty (Belessiotis-Richards et al., 2021; Ma et al., 2020; Emiliano Albanese et al., 2013; Farron et al., 2020; Qiu et al., 2005). A review has indicated that higher blood pressure may increase the risk of dementia in the future, particularly in cases of untreated hypertension (Qiu et al., 2005), potentially aligning with our finding that hypertensive patients are more likely to be assigned to the “Consistently Severe” group.

Our study also found that diabetes was associated with poorer cognition and frailty, which is consistent with a review examining the impact of diabetes on brain function and structure over the past two decades (Moheet et al., 2015). However, prior research on the relationship between diabetes and cognitive function remains inconclusive. For instance, a cross-sectional study in India found that self-reported diabetes is linked to better cognitive performance (Belessiotis-Richards et al., 2021). In addition, a review emphasized that managing diabetes and reducing complications may lower the risk of cognitive impairment, with a stronger association observed in type 2 diabetes patients compared to those with type 1 diabetes (Kodl and Seaquist, 2008).

Furthermore, our study revealed that older adults without complete tooth loss exhibited better cognitive and frailty outcomes, aligning with numerous previous research findings (Hoeksema et al., 2017; Yun et al., 2020; Zhang et al., 2022; Zhang et al., 2022). To reduce the likelihood of tooth loss, various measures can be taken, including early screening for oral health, treatment of periodontal disease, and maintenance of good oral hygiene practices. For older adults experiencing tooth loss, obtaining conventional prostheses as early as possible is advisable to prevent complications arising from the simultaneous exacerbation of cognitive function and frailty resulting from having fewer or no teeth (Yun et al., 2020).

Sensory impairments, such as hearing impairment and vision impairment, are prevalent among older adults. In our study, we found that 17.56% of older adults had hearing impairments, while and even higher proportion, 39.57%, had vision impairments. Age-related hearing loss is associated with changes in both the central and peripheral auditory systems and is characterized by difficulties in understanding words in noisy environments, which may contribute to late-life cognitive disorders (Panza et al., 2018). Similarly, hearing impairment can lead to psychosocial stress, social isolation, and the onset of depression (Johnson et al., 2015), mirroring the mechanisms observed in visual impairments (Reyes-Ortiz et al., 2005; Liljas et al., 2017). Both hearing and vision impairments have been linked to cognitive decline and dementia. Older adults with impaired vision and hearing may face challenges in accessing social support, which is crucial for preventing cognitive decline and frailty. Undetected and untreated impaired vision and hearing can have significant impacts on patients, their loved ones, and society as a whole.

Considering that cognitive decline is irreversible while frailty can be reversed (Baolin et al., 2021), and since both share common trajectories, controlling and intervening in the frailty of older adults can help prevent or slow down their cognitive decline. These findings provide valuable insights for the care of older adults.

Given that many predictors for assigning into the “Consistently Severe” group are preventable, and frailty is reversible, it is crucial to identify older adults at high risk through regular health monitoring and implement interventions as soon as possible. Early detection and intervention can significantly improve the overall health and quality of life for older adults. We recommend that community healthcare centers closely monitor the risk factors identified in the health checks of older adults and identify high-risk populations. By doing so, better management of the health status of them can be achieved, implementing appropriate intervention measures to address these factors. This will be beneficial in delaying or preventing cognitive decline and frailty.

## 5 Strengths and limitations

This study represents a pioneering application of GBDTM to explore the heterogeneity in co-development trajectories of cognitive function and frailty among older adults in China, using longitudinal data. Furthermore, we identified potential factors that increase the likelihood of older adults being classified into the “Consistently Severe” group, characterized by a higher rate of decline in both cognitive function and frailty.

However, it is important to acknowledge the limitations of this study. Firstly, due to data limitation, our assessment of cognitive function focused primarily on immediate and delayed memory, without considering other dimensions such as executive function or orientation function. While it is true that the memory is a highly relevant dimension in relation to dementia, the exclusion of other cognitive dimensions may limit the comprehensive understanding of cognitive function in our findings. Secondly, in our study, several items, particularly those related to chronic diseases, sensory impairment and physical activities, were self-reported by participants. The use of self-reporting introduces the potential for recall bias, as participants may not accurately remember or report their behaviors or conditions. This may impact the validity and reliability of the collected data. However, it is worth noting that self-report of medical conditions has been widely used in studies involving community-dwelling older adults. Additionally, previous research has demonstrated consistency between self-reporting and objective measurements (Yuan et al., 2022; Howrey et al., 2020; Ma et al., 2020; Reyes-Ortiz et al., 2005; Liljas et al., 2017). Despite the limitations of self-reporting, it remains a valuable and commonly employed method for gathering data in this context. Thirdly, it is important to note that while the CHARLS recruited a representative sample in China, it did not include individuals from minority groups residing in regions such as Tibet and Xinjiang. As a result, the findings of this study may not be directly applicable to minority populations. It is recommended that future research should specifically investigate the co-development trajectories of cognition and frailty among these minority populations to ensure a more comprehensive understanding of the topic.

## 6 Conclusion

In this longitudinal study, one group showed better cognitive function and lower levels of frailty, while the “Consistently Severe” group exhibited poorer cognitive function and higher levels of frailty. Several characteristics were associated with being in the “Consistently Severe” group, including being female, older, illiterate, residing in rural areas, and being unmarried. Additionally, individuals in this group reported worse overall health and a higher prevalence of conditions such as diabetes, hypertension, hearing impairment, vision impairment, and complete tooth loss.

While cognitive impairment may be irreversible, frailty is a reversible condition. Therefore, early detection of frailty is crucial for preventing cognitive decline, given their share trajectory. Furthermore, many predictors for being in the “Consistently Severe” group are preventable, highlighting the importance of regular health monitoring and timely interventions.

Community healthcare centers should closely monitor the risk factors identified in the annual health checks for older adults. This enables the development of targeted interventions to address these factors, which may help delay or prevent cognitive decline and frailty.

## Data Availability

Publicly available datasets were analyzed in this study. This data can be found at: http://charls.pku.edu.cn/en.
