# Supplementary material for: Trajectories of cognitive function and frailty in older adults in China: a longitudinal study
Source: Front Aging Neurosci. 2024 Nov 14;16:1465914. doi: 10.3389/fnagi.2024.1465914 (PMC11602512; doi:10.3389/fnagi.2024.1465914)
Supplement: Supplementary file 1 [file Table_1.docx]

**Supplemental Material**

| **List of Supplementary Table** | |
| --- | --- |
| **Number** | **Title** |
| Supplementary Table 1 | Coding of Frailty |
| Supplementary Table 2 | Definition and coding of analyzed variables |
| Supplementary Table 3 | Estimates of dual trajectories of cognitive function and frailty |
| Supplementary Table 4 | Associated factors of trajectories of cognitive function |
| Supplementary Table 5 | Associated factors of trajectories of frailty |

**Supplementary Table1 Coding of Frailty**

| **Dimension** | **Item** | **Assignment** |
| --- | --- | --- |
| Physical Functioning Limitation | Do you have difficulty running or jogging 1 kilometer? | 0=No difficulty;  0.33=Difficulty but can still complete;  0.67=Difficulty, need assistance;  1=Unable to complete |
|  | Do you have difficulty walking 1 kilometer? |  |
|  | Do you have difficulty walking 100 meters? |  |
|  | Do you have difficulty standing up after sitting for a long time in a chair? |  |
|  | Do you have difficulty climbing several flights of stairs continuously? |  |
|  | Do you have difficulty bending, squatting, or kneeling? |  |
|  | Do you have difficulty raising your arms above your shoulders? (No difficulty if both arms are fine, otherwise considered as having difficulty) |  |
|  | Do you have difficulty lifting a 10-pound object? |  |
|  | Do you have difficulty picking up a small coin from a table? |  |
|  |  |  |
| Chronic Disease | Hypertension | 0=No; 1=Yes |
|  | Abnormal Blood Lipids |  |
|  | Diabetes |  |
|  | Chronic Lung Disease |  |
|  | Liver Disease |  |
|  | Heart Disease |  |
|  | Kidney Disease |  |
|  | Stomach Disease |  |
|  | Emotional and Mental Health Issues |  |
|  | Memory-related Diseases |  |
|  | Arthritis or Rheumatism |  |
|  | Asthma |  |
|  |  |  |
| Activities of Daily Living (ADL) | Do you have difficulty dressing yourself due to health and memory reasons? | 0=No difficulty;  0.33=Difficulty but can still complete;  0.67=Difficulty, need assistance;  1=Unable to complete |
|  | Do you have difficulty bathing due to health and memory reasons? |  |
|  | Do you have difficulty feeding yourself, such as using utensils, due to health and memory reasons? |  |
|  | Do you have difficulty getting in and out of bed? |  |
|  | Do you have difficulty using the toilet, including squatting and standing up, due to health and memory reasons? |  |
|  | Do you have difficulty controlling your bowel and bladder movements due to health and memory reasons? |  |
|  |  |  |
| Instrumental Activity of Daily Living (IADL) | Do you have difficulty performing household chores due to health and memory reasons? | 0=No difficulty;  0.33=Difficulty but can still complete;  0.67=Difficulty, need assistance;  1=Unable to complete |
|  | Do you have difficulty cooking due to health and memory reasons? |  |
|  | Do you have difficulty going to the store to buy groceries due to health and memory reasons? |  |
|  | Do you have difficulty managing your medication independently? (This includes remembering when to take your medication and how much to take.) |  |
|  | Do you have difficulty managing finances due to health and memory reasons, such as paying bills, keeping track of expenses, and managing your possessions? |  |
|  |  |  |
| Mental Health | Feeling down  Feeling like everything is an effort  Feeling fearful  Feeling happy  Feeling like can't continue with my life | 0=Very rarely or almost never (<1 day);  0.33=Not too much (1-2 days);  0.67=Sometimes or about half the time (3-4 days);  1=Most of the time (5-7 days). |
|  |  |  |
| Health Condition | How would you rate your health condition? Is it very good, good, fair, poor, or very poor? | 0=Very good;  0.25=Good;  0.50=Fair;  0.75=Poor;  1=Very poor |

**Supplementary Table 2. Definition and coding of analyzed variables**

| **Variables** | **Definition of variables** |
| --- | --- |
| Cognitive score | Continuous variable |
| Frailty index | Continuous variable |
| Gender | 0 = male, 1 = female |
| Age | 1 = 55~64 years old, 2 = 65~74 years old, 3 = 75 years old or older |
| Residential area | 0 = rural, 1 = urban |
| Marital status | 0 = non-married, 1 = married |
| Education | 0 = illiterate, 1 = literate |
| Annual personal expenditure | 0 = lower than the average in 2010, 1 = higher than the average in 2010 |
| Drinking | 1 = current, 2 = previous, 3 = no |
| Hypertension  Diabetes | 0 = no, 1 = yes |
| Vision impairment  Hearing impairment  Complete tooth loss | 0 = no, 1 = yes |

**Supplementary Table 3 Estimates of dual trajectories of cognitive function and frailty**

| **Dual trajectory** | **Group 1** |  | **Group 2** |  |
| --- | --- | --- | --- | --- |
| Equation | Estimates | SE | Estimates | SE |
| Cognitive Score |  |  |  |  |
| Intercept | 5.84*** | 0.07 | 7.21*** | 0.05 |
| Linear slope | -0.30*** | 0.02 | -0.29*** | 0.03 |
| Quadratic slope | - | - | 0.02 | 0.00 |
| Frailty Index |  |  |  |  |
| Intercept | 0.24*** | 0.00 | 0.09*** | 0.00 |
| Linear slope | 0.01*** | 0.00 | 0.00* | 0.00 |
| Quadratic slope | 0.00* | 0.00 | 0.00*** | 0.00 |

**p* < 0.05.; ***p* < 0.01.; ****p* < 0.001

**Supplementary Table 4 Associated factors of trajectories of cognitive function**

| **Variables** | **Group 2/Group1 (ref)** | |
| --- | --- | --- |
|  | ***OR*** | **95% *CI*** |
| Age |  |  |
| 55~64 (ref) |  |  |
| 65~74 | 2.10*** | 1.87 – 2.36 |
| ≥75 | 4.15*** | 3.22 – 5.40 |
| Education |  |  |
| Illiterate (ref) |  |  |
| Literate | 0.19*** | 0.17 – 0.22 |
| Gender |  |  |
| Male (ref) |  |  |
| Female | 0.95 | 0.84 – 1.07 |
| Residence |  |  |
| Rural (ref) |  |  |
| Urban | 0.53*** | 0.47 – 0.59 |
| Average Household Annual Expenditure |  |  |
| Low (ref) |  |  |
| High | 0.83* | 0.70 – 0.98 |
| Marriage |  |  |
| Unmarried (ref) |  |  |
| Married | 0.72*** | 0.61 – 0.84 |
| Drinking |  |  |
| Still Drinking (ref) |  |  |
| Abstinence | 0.82 | 0.66 – 1.01 |
| Never Drinking | 0.97 | 0.85 – 1.10 |
| Hypertension |  |  |
| No (ref) |  |  |
| Yes | 0.98 | 0.87 – 1.06 |
| Diabetes |  |  |
| No (ref) |  |  |
| Yes | 0.87 | 0.70 – 1.06 |
| Complete tooth loss |  |  |
| No (ref) |  |  |
| Yes | 1.19* | 1.00 – 1.42 |
| Vision impairment |  |  |
| No (ref) |  |  |
| Yes | 1.40*** | 1.26 – 1.56 |
| Hearing impairment |  |  |
| No (ref) |  |  |
| Yes | 1.51*** | 1.31 – 1.73 |

Notes: *OR* = odds ratio; 95% *CI* = 95% confidence interval; ref = reference group;

**p* < 0.05; ***p* < 0.01; ****p* < 0.001.

**Supplementary Table 5 Associated factors of trajectories of frailty**

| **Variables** | **Group 2/Group1 (ref)** | |
| --- | --- | --- |
|  | ***OR*** | **95% *CI*** |
| Age |  |  |
| 55~64 (ref) |  |  |
| 65~74 | 1.61*** | 1.42 – 1.83 |
| ≥75 | 1.49*** | 1.18 – 1.87 |
| Education |  |  |
| Illiterate (ref) |  |  |
| Literate | 0.81*** | 0.71 – 0.92 |
| Gender |  |  |
| Male (ref) |  |  |
| Female | 1.58*** | 1.38 – 1.81 |
| Residence |  |  |
| Rural (ref) |  |  |
| Urban | 0.65*** | 0.57 – 0.73 |
| Average Household Annual Expenditure |  |  |
| Low (ref) |  |  |
| High | 1.13 | 0.94 – 1.35 |
| Marriage |  |  |
| Unmarried (ref) |  |  |
| Married | 0.81** | 0.69 – 0.95 |
| Drinking |  |  |
| Still Drinking (ref) |  |  |
| Abstinence | 0.94 | 0.72 – 1.23 |
| Never Drinking | 1.52*** | 1.30 – 1.78 |
| Hypertension |  |  |
| No (ref) |  |  |
| Yes | 2.63*** | 2.33 – 2.96 |
| Diabetes |  |  |
| No (ref) |  |  |
| Yes | 2.55*** | 2.08 – 3.13 |
| Complete tooth loss |  |  |
| No (ref) |  |  |
| Yes | 1.33*** | 1.12 – 1.58 |
| Vision impairment |  |  |
| No (ref) |  |  |
| Yes | 2.03*** | 1.81 – 2.28 |
| Hearing impairment |  |  |
| No (ref) |  |  |
| Yes | 1.65*** | 1.44 – 1.90 |

Notes: *OR* = odds ratio; 95% *CI* = 95% confidence interval; ref = reference group;

**p* < 0.05.; ***p* < 0.01.; ****p* < 0.001.
